# A Panel of Synapse-Related Genes as a Biomarker for Gliomas

**DOI:** 10.3389/fnins.2020.00822

**Published:** 2020-08-11

**Authors:** Xiangwen Ji, Hongwei Zhang, Qinghua Cui

**Affiliations:** ^1^Department of Biomedical Informatics, Center for Non-coding RNA Medicine, MOE Key Lab of Cardiovascular Sciences, School of Basic Medical Sciences, Peking University, Beijing, China; ^2^Department of Physiology and Pathophysiology, Center for Non-coding RNA Medicine, MOE Key Lab of Cardiovascular Sciences, School of Basic Medical Sciences, Peking University, Beijing, China; ^3^Department of Neurosurgery, Sanbo Brain Hospital, Capital Medical University, Beijing, China

**Keywords:** glioma, synapse, biomarker, survival, WHO grade

## Abstract

Gliomas are the most common primary brain cancers. In recent years, *IDH* mutation and 1p/19q codeletion have been suggested as biomarkers for the diagnosis, treatment, and prognosis of gliomas. However, these biomarkers are only effective for a part of glioma patients, and thus more biomarkers are still emergently needed. Recently, an electrochemical communication between normal neurons and glioma cells by neuro-glioma synapse has been reported. Moreover, it was discovered that breast-to-brain metastasis tumor cells have pseudo synapses with neurons, and these synapses were indicated to promote tumor progression and metastasis. Based on the above observations, we first curated a panel of 17 synapse-related genes and then proposed a metric, synapse score to quantify the “stemness” for each sample of 12 glioma gene expression datasets from TCGA, CGGA, and GEO. Strikingly, synapse score showed excellent predictive ability for the prognosis, diagnosis, and grading of gliomas. Moreover, being compared with the two established biomarkers, *IDH* mutation and 1p/19q codeletion, synapse score demonstrated independent and better predictive performance. In conclusion, this study proposed a quantitative method, synapse score, as an efficient biomarker for monitoring gliomas.

## Introduction

Brain and other nervous system cancers are estimated to take up 1.4% of new cancers but 2.9% of cancer deaths in 2019 (Brain and Other Nervous System Cancer, 2019). Gliomas are the most frequent of these cancers, including astrocytoma (including glioblastoma), oligodendroglioma, ependymoma, oligoastrocytoma (mixed glioma), malignant glioma, not otherwise specified (NOS) glioma, and a few rare histologies (Ostrom et al., 2016). The World Health Organization (WHO) classified gliomas into grades I to IV and introduced biomarkers of *IDH* mutation and 1p/19q codeletion in the 2016 edition (Louis et al., 2007; Wesseling and Capper, 2018). Glioblastoma (WHO grade IV) accounts for about half of gliomas, with a median survival of less than 2 years (Gramatzki et al., 2016; Ostrom et al., 2016). Gliomas with lower grade have a diverse prognosis, either progressing to be as poor as glioblastoma or living more than 10 years after effective treatment (Ruda and Soffietti, 2017).

Over the years, with the fast improvement of omics and big data technology, RNA sequencing has been developing toward lower cost and higher throughput, producing a large amount of biological and medical data, which provides great convenience for life science research (Bolouri et al., 2016). Impelled by advantage of big data analysis, numerous biomarkers have been found in the diagnosis and prognosis of gliomas (Kros et al., 2015). Gene set enrichment analysis (GSEA) provides a facility to extract effective information from a large number of RNA expression data (Subramanian et al., 2005). Moreover, single sample GSEA (ssGSEA) can calculate without group information and give every sample an enrichment score (Barbie et al., 2009). The Biomarkers such as *IDH* mutation and 1p/19q codeletion provided help for monitoring the development and prognosis of gliomas but are only effective for a part of patients (Aibaidula et al., 2017). Therefore, given the enormous severity of gliomas, more biomarkers are emergently needed.

It is recently reported that neuron and glioma have electrochemical communication through AMPA receptor-dependent synapses between presynaptic neurons and postsynaptic glioma cells (Venkataramani et al., 2019; Venkatesh et al., 2019). These observations suggest that the neural synaptic electrochemical connections promote glioma progression. Simultaneously, an appearance of glutamatergic “pseudo-tripartite” synapses between breast-to-brain metastasis tumor cells and neurons was observed (Zeng et al., 2019). Based on these anatomical and cytological findings, we hypothesized that the synapse-related genes can be used as a biomarker for glioma prognosis. To confirm this hypothesis, here we first curated a list of genes involved in synapse-related functions and then performed ssGSEA analysis for glioma gene expression datasets from the Cancer Genome Atlas (TCGA), the Chinese Glioma Genome Atlas (CGGA), and the Gene Expression Omnibus (GEO). Strikingly, these synapse-related genes were found to be an independent and effective biomarker for gliomas.

## Materials and Methods

### Gene Expression Datasets and Analysis

RNAseq data, normalized in fragments per kilo-base per million mapped fragments, as well as sample and clinical information were obtained from TCGA data portal^1^. WHO grade, *IDH* mutation status, and 1p/19q codeletion status were obtained from the study by Ceccarelli et al. (2016). CGGA^2^ provides tumor gene expression data for thousands of glioma patients (including one microarray and two RNAseq batches), as well as corresponding clinical data. The calculation and presentation of the results will be conducted separately due to different platforms and batches. In addition, glioma microarray gene expression profiling data (GSE4290, GSE16011, GSE50161, GSE52009, GSE54004, GSE61374, and GSE107850) were available at GEO datasets^3^. Gene expression data were structured with gene symbols as row names and sample IDs as column names; duplicate gene symbols were averaged using their median value.

### Synapse-Related Genes Screening

Gene ontology (GO) terms, which were related to synapse, neuron, neurotransmitter transport, glutamate receptor, or cell junction, were selected from NCBI^4^. Using ssGSEA, we calculated enrichment scores (ESs) for each GO term and each sample in two CGGA RNAseq batches. The ssGSEA algorithm was performed by python (v3.6.8) package gseapy (v0.9.13), which is a python wrapper for GSEA and ssGSEA. The minimum number of genes in the gene set was set as 10, and the maximum was 1,000. As a result, 163 of 581 terms were retained. Cox regression models were used to calculate the hazard ratios (HRs) and *p*-values for ESs of each GO term. We used CoxPHFitter from python package lifelines (v0.23.7) to fit Cox models. Default parameters were used except the data frame and the column names of survival times and events. *P*-values were adjusted using Benjamini-Hochberg method. The false discovery rates (FDRs) of the two batches are multiplied to calculate the combined FDR (Supplementary File S1). The terms with different directions in two batches (HR < 1 in one batch and HR > 1 in the other) were excluded. Terms with top 10 smallest combined FDR values, except “peripheral nervous system neuron development” (GO:0048935) as gliomas are located in the central nervous system, are used for subsequent analysis (ionotropic glutamate receptor signaling pathway, AMPA glutamate receptor complex, regulation of short-term neuronal synaptic plasticity, dopaminergic synapse, synapse maturation, excitatory postsynaptic potential, parallel fiber to Purkinje cell synapse, synapse organization, and regulation of AMPA receptor activity). Next, we evaluated the HRs and *p*-values of 171 genes (eight genes are not in the datasets) from these nine GO terms and calculated the combined FDRs (Supplementary File S2). One hundred forty-four genes were filtered out with the same directions in two data batches. Then we obtained ESs of genes with top *n* (*n* = 1, 2, …, 144) smallest combined FDRs for each sample in two data batches. After we evaluated the combined FDRs of every gene set, the gene set with the top 17 genes were selected (Supplementary File S3). Finally, using this synapse-related 17-gene set, we performed ssGSEA (default parameters) and calculated ESs for samples of TCGA, CGGA, and GEO datasets. We defined the ES as synapse score.

### Statistical Analysis

Kaplan–Meier (K–M) curves and Cox proportional hazards regression were performed by R packages survival (v2.44-1.1) and survminer (v0.4.6) and python package lifelines (v0.23.7). Log rank test was used to calculate the difference between two K–M curves. Significance of difference between two groups of continuous variables was analyzed by two-sided Wilcoxon rank sum test. Receiver operating characteristic (ROC) curve and area under ROC curve (AUROC) were processed by R package pROC (Robin et al., 2011) (v1.15.3). All statistical significances above were calculated by R (v3.5.2). Spearman’s correlation analysis was applied to evaluate the correlation using python package scipy (v1.2.1). *P*-values < 0.05 were considered significant.

## Results

### The Screening of Synapse-Related Genes

**FIGURE 1:**
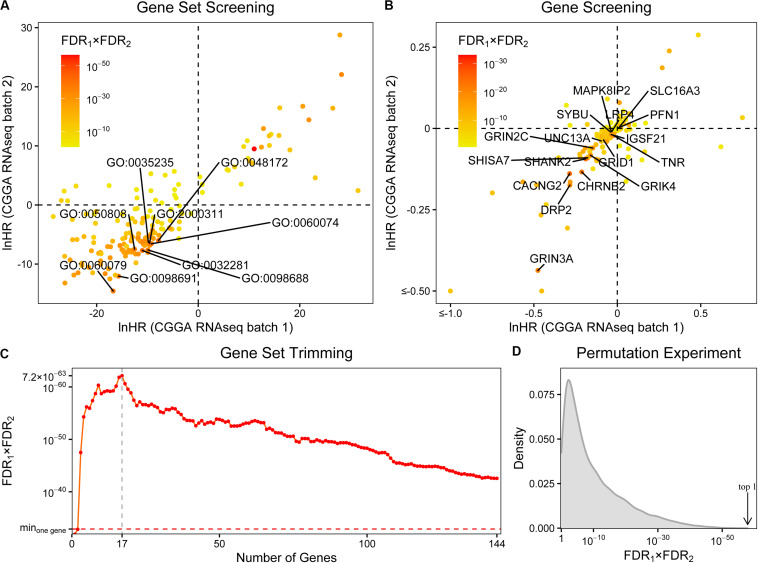
The screening of synapse-related genes. **(A)** The survival prognostic performances of 163 synapse-related gene ontology terms. The chosen terms were labeled. **(B)** The prognostic performances of 171 collected genes. The finally selected genes were labeled. **(C)** The prognostic performances of gene sets with different sizes. Min_*one gene*_: the best performance of single collected gene. The prognostic performances were evaluated by hazard ratios (HRs) of Cox regression. FDRs were calculated by Benjamini-Hochberg method. **(D)** The distribution of combined FDRs of 10,000 random 17-gene sets. Arrow: the combined FDR of our synapse-related 17-gene set.

In order to investigate whether synapse-related genes can be biomarkers for glioma patients, we first curated a list of GO terms associated with synapse, neuron, neurotransmitter transport, glutamate receptor, or cell junction. After excluding the terms with less than 10 or more than 1,000 genes, 163 terms were retained. Then we evaluated the survival prediction performances of these gene sets in two CGGA RNAseq batches using ssGSEA and Cox regression (Supplementary File S1). Most (118/163) of the terms were found to have HR < 1 in both data batches. The 10 best performed terms were further selected, and “peripheral nervous system neuron development” (GO:0048935) is excluded as gliomas are located in the central nervous system (Figure 1A). As a result, 171 genes were collected.

Afterward, we assessed the prognostic performances of these genes using Cox regression (Figure 1B and Supplementary File S2). 144 genes were filtered out with the same directions (both HRs < 1 or both HRs > 1) in two data batches. To further trim the gene set, we calculated ESs of gene sets which include the top *n* (*n* = 1, 2, …, 144) best performed genes and evaluated their survival prognostic abilities (Figure 1C and Supplementary File S3). In most (142/144) cases, the gene sets performed better than any of the 144 genes on its own. Finally, the gene set with 17 genes was selected (Figure 1B and Supplementary Table S1), including profilin 1 (*PFN1*), SH3 and multiple ankyrin repeat domains 2 (*SHANK2*), calcium voltage-gated channel auxiliary subunit gamma 2 (*CACNG2*), tenascin R (*TNR*), shisa family member 7 (*SHISA7*), cholinergic receptor nicotinic beta 2 subunit (*CHRNB2*), glutamate ionotropic receptor NMDA type subunit 3A (*GRIN3A*), mitogen-activated protein kinase eight interacting protein 2 (*MAPK8IP2*), glutamate ionotropic receptor delta type subunit 1 (*GRID1*), unc-13 homolog A (*UNC13A*), LDL receptor-related protein 4 (*LRP4*), syntabulin (*SYBU*), solute carrier family 16 member 3 (*SLC16A3*), dystrophin-related protein 2 (*DRP2*), glutamate ionotropic receptor kainate type subunit 4 (*GRIK4*), glutamate ionotropic receptor NMDA type subunit 2C (*GRIN2C*), and immunoglobin superfamily member 21 (*IGSF21*).

As the next few gene sets, neurotransmitter uptake (GO:0001504), glutamate receptor signaling pathway (GO:0007215), NMDA selective glutamate receptor complex (GO:0017146), and glutamate receptor activity (GO:0008066) have combined FDRs of similar magnitudes (<5 × 10^–35^); the choice of top 10 terms could be too arbitrary. It may be useful to include them in subsequent analyses. We took these terms into consideration one by one and performed the same steps of screening and trimming described above. The inclusion of the term neurotransmitter uptake did not change the final result, and the same 17 genes were screened out. As for the other three terms, they all resulted in a 20-gene set, adding potassium voltage-gated channel subfamily B member 1 (*KCNB1*), nicastrin (*NCSTN*), and phospholipase C beta 1 (*PLCB1*) to the previous 17-gene set. But its combined *p*-value (2.38 × 10^–64^) was a little worse than the previous 17-gene set (3.70 × 10^–66^). Although there are still many significant terms, like focal adhesion (GO:0005925) at #15, we could not consider more due to the time complexity of subsequent screening and trimming. Finally, we decided to use the 17-gene set for future validations.

To further verify the efficiency of the 17-gene set, a permutation experiment was performed. After randomly selecting 10,000 sets with 17 genes from all the 23,271 genes that exist in both batches of datasets, we tested their prognostic abilities by ssGSEA and Cox regression. As a result, the combined FDR of the selected 17-gene set ranked first in all random gene sets ascendingly (Figure 1D and Supplementary File S4).

### The Panel of Synapse-Related Genes Serves as a Novel Biomarker for Gliomas

**FIGURE 2:**
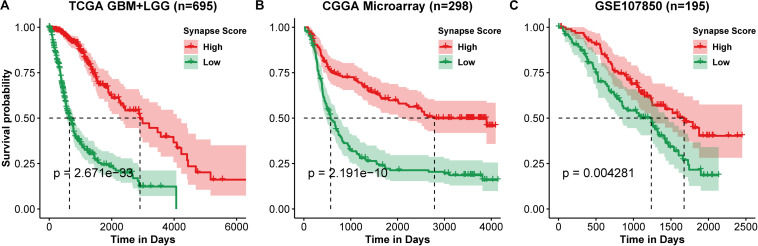
Kaplan–Meier curve of overall survival. **(A)** TCGA lower grade glioma (LGG) and glioblastoma multiforme (GBM). **(B)** CGGA Microarray. **(C)** GSE107850 from GEO datasets. Group was separated by the median value of synapse scores. Differences between two curves were estimated by log-rank test.

**FIGURE 3:**
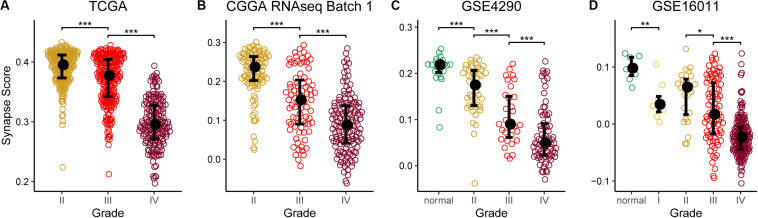
Synapse scores were significantly lower in higher grade gliomas. **(A)** TCGA lower grade glioma (LGG) and glioblastoma multiforme (GBM). **(B)** CGGA RNAseq batch 1. **(C)** GSE4290. **(D)** GSE16011. Significances of difference between two groups were analyzed by two-side Wilcoxon rank sum test. *p < 0.05, **p < 0.01, ***p < 0.001.

Using the 17 collected synapse-related genes, we performed ssGSEA to TCGA, CGGA, and GEO datasets, and the ESs, defined as synapse score, were used for survival analysis. The results show that glioma patients with higher synapse scores have longer overall survival time (Figure 2). Cox regression analysis also shows the same results (Table 1). Moreover, patients with higher WHO grade have significantly lower synapse scores (Figure 3 and Supplementary Figure S1), which agrees with the survival analysis. In addition, it is worthy to mention that there were normal brain samples in datasets GSE4290 (Figure 3C), GSE16011 (Figure 3D), and GSE50161 (Supplementary Figure S1c). The synapse scores of normal samples were significantly higher than glioma samples, suggesting that the synapse score shows an ability to distinguish between glioma and normal brain tissue by giving a cutoff value, which reveals a potential diagnostic application of synapse score. ROC analyses were further used to evaluate the diagnostic ability; the areas under the curve (AUCs) of GSE4290, GSE16011, and GSE50161 datasets are 0.89, 0.94, and 0.99, respectively (Supplementary Figure S2).

**TABLE 1:** The predictive ability of synapse score adjusted using WHO grade, *IDH* mutation, and 1p/19q codeletion.

Datasets	Hazard ratio (95% CI) (no. of samples)			
	Unadjusted	Grade-adjusted	*IDH* Status-adjusted	1p/19q Codeletion-adjusted
CGGA Microarray	0.0379 (0.0151–0.0947)*** (*n* = 298)	0.270 (0.0852–0.856)* (*n* = 295)	0.0645 (0.0249–0.167)*** (*n* = 296)	0.0491 (9.04 × 10^–3^ to 0.267)*** (*n* = 91)
CGGA RNAseq batch 1	5.77 × 10^–5^ (1.15 × 10^–5^ to 2.89 × 10^–4^)*** (*n* = 311)	1.04 × 10^–3^ (1.57 × 10^–4^ to 6.85 × 10^–3^)*** (*n* = 307)	2.97 × 10^–5^ (3.49 × 10^–6^ to 2.52 × 10^–4^)*** (*n* = 310)	2.93 × 10^–4^ (4.61 × 10^–5^ to 1.86 × 10^–3^)*** (*n* = 303)
CGGA RNAseq batch 2	3.31 × 10^–4^ (9.18 × 10^–5^ to 1.20 × 10^–3^)*** (*n* = 619)	9.17 × 10^–3^ (1.99 × 10^–3^ to 0.0423)*** (*n* = 619)	5.62 × 10^–3^ (1.16 × 10^–3^ to 0.0272)*** (*n* = 574)	7.85 × 10^–4^ (1.72 × 10^–4^ to 3.58 × 10^–3^)*** (*n* = 555)
TCGA GBM + LGG	2.84 × 10^–7^ (4.00 × 10^–8^ to 2.02 × 10^–6^)*** (*n* = 695)	8.18 × 10^–3^ (4.13 × 10^–4^ to 0.162)** (*n* = 634)	4.83 × 10^–3^ (2.96 × 10^–4^ to 0.0788)*** (*n* = 685)	9.48 × 10^–7^ (1.03 × 10^–7^ to 8.77 × 10^–6^)*** (*n* = 688)
GSE107850	1.53 × 10^–4^ (2.20 × 10^–6^ to 0.0107)*** (*n* = 195)		3.09 × 10^–4^ (3.03 × 10^–6^ to 0.0314)*** (*n* = 180)	

Hazard ratio (HR) and 95% confidence interval (95% CI) of synapse score using univariate and multivariate Cox proportional hazards regression models for gliomas were shown. HR with 95% CI that does not include one is considered significant. *p < 0.05, **p < 0.01, ***p < 0.001.

### Comparison of Synapse Score With Established Biomarkers

*IDH* mutation and 1p/19q codeletion are two established biomarkers for gliomas. Both biomarkers provided great help for monitoring glioma development, but both are effective on only some patients. Therefore, it is interesting to explore whether synapse score is an independent biomarker and whether synapse score is better than the established biomarkers or not. For doing so, we first analyzed the relationship of synapse scores with *IDH* mutation and 1p/19q codeletion status. We found that *IDH*-mut gliomas were associated with significantly higher synapse scores than *IDH*-wt ones (Supplementary Figures S3a–d). And 1p/19q codeletion gliomas represent higher synapse scores than non-codeletion ones (Supplementary Figures S3e–h). Moreover, after removing the effects of the two established biomarkers using multivariate Cox regression model, we revealed that synapse score is an independent biomarker for predicting prolonged overall survival in gliomas (Table 1). In addition, the grading ability of synapse score is also independent of *IDH* mutation and 1p/19q codeletion (Supplementary Figure S4). Finally, we compared the survival predictive performance of synapse score, *IDH* mutation, and 1p/19q codeletion status (Supplementary Table S2). In most instances, synapse score outperforms *IDH* mutation and 1p/19q codeletion.

## Discussion

Given the recently revealed roles of neuro-glioma synapse in glioma development, here we curated a panel of 17 synapse-related genes and proposed the synapse score as a biomarker for the prognosis, grading, and diagnosis of gliomas. The synapse score was validated by more than 3,000 samples of 12 datasets from TCGA, CGGA, and GEO.

AMPA (α-amino-3-hydroxy-5-methyl-4-isoxazole propionic acid) receptor, one type of glutamate receptors, was focused on in recent studies of neuron-glioma synapses (Venkataramani et al., 2019; Venkatesh et al., 2019). In our study, several AMPA glutamate receptor-related terms, such as ionotropic glutamate receptor signaling pathway (GO:0035235), AMPA glutamate receptor complex (GO:0032281), and regulation of AMPA receptor activity (GO:2000311), were filtered out to have strong survival predictive capacities, suggesting a significant role of AMPA receptor in gliomas.

In addition to AMPA receptor, other ionotropic glutamate receptor genes are also used in the 17-gene set, including N-Methyl-D-aspartate (NMDA) receptor [*GRIN3A* (Marco et al., 2013), *GRIN2C* (Collingridge et al., 2009)], kainate receptor [*GRIK4* (Arora et al., 2018)], and non-classical glutamate receptor such as glutamate delta-1 receptor [*GRID1* (Gupta et al., 2015)], suggesting that other ionotropic glutamate receptors also perform important functions in gliomas. Meanwhile, a gene from other synaptic receptors such as nicotinic acetylcholine receptor [*CHRNB2* (Diaz-Otero et al., 2008)] was also collected. More genes do not belong to receptors, and they perform neuronal-specific synthesis and glycosylation [*TNR* (Woodworth et al., 2004)], signal transduction [*MAPK8IP2* (Kennedy et al., 2007)], neurotransmission [*UNC13A* (Reddy-Alla et al., 2017), *LRP4* (Sun et al., 2016)], synapse formation [*LRP4* (Karakatsani et al., 2017)], inhibitory synapse differentiation [*IGSF21* (Tanabe et al., 2017)], and other functions in synapses (Supplementary Table S1).

Many of the selected genes have been found to be associated with neurological diseases, including autism [*SHANK2* (Monteiro and Feng, 2017; Won et al., 2012)], chronic pain [*CACNG2* (Bortsov et al., 2019; Nissenbaum et al., 2010)], epilepsy [*CHRNB2* (Diaz-Otero et al., 2008)], Huntington’s disease [*GRIN3A* (Marco et al., 2013)], and neurodegenerative diseases [*SYBU* (Bereczki et al., 2018)] (Supplementary Table S1). However, only a few genes have been studied in gliomas. For example, *PFN1* has been found to be involved in tumor angiogenesis in glioblastoma (Fan et al., 2014) and was also found to be associated with poor prognosis in our study (HR > 1) (Figure 1B). According to a proteomics study of gliomas (Bi et al., 2017), *TNR* is down-regulated in glioblastomas. A similar result was found in our study, that low expression of this gene was correlated with poor prognosis (HR < 1). These studies validate our findings and suggest the research and application values of other synapse-related genes in gliomas.

When screening GO terms, there are 17 terms with the opposite directions (HR > 1 in one batch and HR < 1 in the other). Interestingly, all of these terms are negative in the batch 1 dataset and positive in the batch two dataset. There are 4 terms that are not significant in both datasets (FDR ≥ 0.05), which may be random effects. In addition, 10 terms are only significant in batch one, while one term is only significant in batch two, which may be caused by batch effect and differences of samples. Moreover, there are two terms that are significant but have opposite directions in two batches (neuroblast proliferation and neuron maturation). Given their low FDR ranking (FDR1: 140th, FDR2: 126th for neuroblast proliferation, FDR1: 144th, FDR2: 121st for neuron maturation out of 163 terms, ascendingly), these could be false positives. The practical effects of these terms need to be widely validated in future studies.

There are 121 significant (FDR1 < 0.05 and FDR2 < 0.05) synapse-related terms with the same direction of HRs in two batches of datasets, suggesting important roles of synapse-related genes in gliomas. But we could not consider all of the terms and genes due to time complexity. Finding the best gene set is a non-deterministic polynomial-time (NP) hard problem. In this paper, we used heuristic algorithms to find the optimal gene set by adding genes one by one in ascending order of combined FDR. It is known that heuristic algorithms do not always get the best results. There could be a gene set and a machine learning method with better prognostic ability using the synapse-related genes. Although our 17-gene set may not be the best result, it is still validated by a permutation experiment and 10 additional datasets and showed better prognostic capability than traditional biomarkers, *IDH* mutation, and 1p/19q codeletion, revealing the extensive research and application value of synapse-related genes in gliomas.

In spite of its ability as glioma biomarker for the identified synapse-related gene panel, it should be especially noted that the result seems the opposite of existing knowledge. That is, it was reported that neuro-glioma synapse could promote tumor progression and metastasis (Venkataramani et al., 2019; Venkatesh et al., 2019; Zeng et al., 2019), which thus can infer that synapse-related genes should result in a poorer prognosis. However, we revealed it is associated with a better but not poorer prognosis. One possible reason is that the more severe the disease is, the less the normal neurons exist. Molecular processes may play different roles in various cells, organs, and diseases. For example, as an important discovery in glioma research, *IDH* mutation is identified as one of the early events of gliomas, and the epigenetic changes caused by *IDH* mutation are considered as a main tumor driver (Turkalp et al., 2014). Nevertheless, clinical studies have found that *IDH* mutation can lead to a longer survival time (Cancer Genome Atlas Research Network et al., 2015). Similarly, immunotherapy, which has been widely used, was criticized for producing serious side effects (Moslehi et al., 2018). These instances suggest that the synapse-related gene panel could also have multiple aspects.

Analogously, *IDH*-mut and 1p/19q codeletion are typically biomarkers that promote glioma progression but benefit prognosis. Existing studies have focused on mechanisms that promote glioma, but the reasons for better prognosis are generally reported by clinical studies, such as better chemoradiotherapy sensitivity (Chen et al., 2017). We conjectured that synapses, *IDH* mutation, and 1p/19q codeletion shared a part of the mechanism that resulted in the observed phenomenon. The causations in synapses, mutations, and gliomas remain to be explored.

In summary, although the mechanism is unclear, we revealed that the proposed synapse score is an independent and potentially better biomarker for glioma overall survival and shows a predictive capacity in different grade gliomas and normal brain tissues, which could be useful in the prognosis, grading, and diagnosis of gliomas.

## Data Availability Statement

Publicly available datasets were analyzed in this study. This data can be found here: https://portal.gdc.cancer.gov/, http://firebrowse.org/, http://www.cgga.org.cn/, and https://www.ncbi.nlm.nih.gov/gds/.

## Author Contributions

QC conceived the project. XJ performed the analysis and conducted the experiments. XJ, HZ, and QC wrote the manuscript. All authors contributed to the article and approved the submitted version.

## Conflict of Interest

The authors declare that the research was conducted in the absence of any commercial or financial relationships that could be construed as a potential conflict of interest.

## Footnotes

1 https://portal.gdc.cancer.gov/

2 http://www.cgga.org.cn/

3 https://www.ncbi.nlm.nih.gov/gds/

4 https://ftp.ncbi.nih.gov/gene/DATA/
