# Supplementary material for: A Panel of Synapse-Related Genes as a Biomarker for Gliomas
Source: Front Neurosci. 2020 Aug 11;14:822. doi: 10.3389/fnins.2020.00822 (PMC7431624; doi:10.3389/fnins.2020.00822)
Supplement: Supplementary file 1 [file Image_1.PDF]

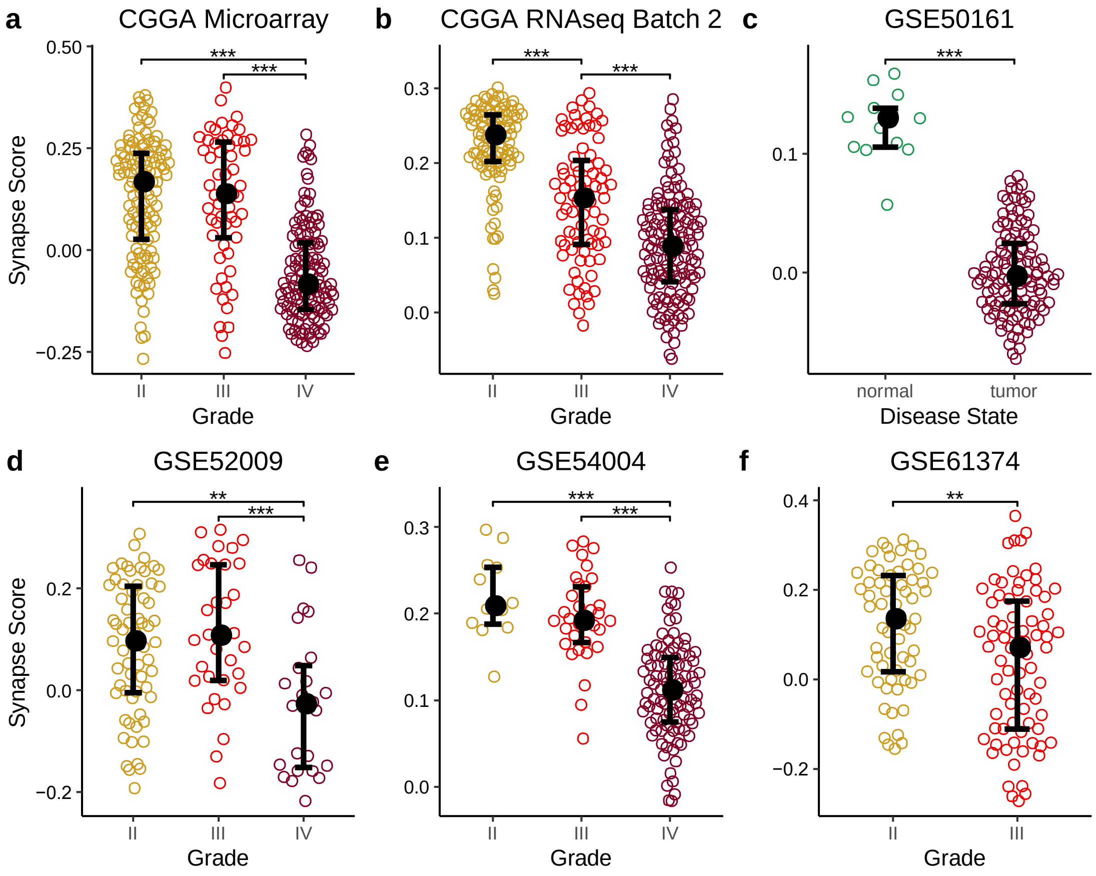

Supplementary Figure S1 Synapse scores were significantly lower in higher grade gliomas. (a) CGGA Microarray. (b) CGGA RNAseq batch 2. (c) GSE50161. (d) GSE52009. (e) GSE54004. (f) GSE61374. Significances of difference between two groups were analyzed by two-side Wilcoxon rank sum test. * p<0.05, ** p<0.01, *** p<0.001.

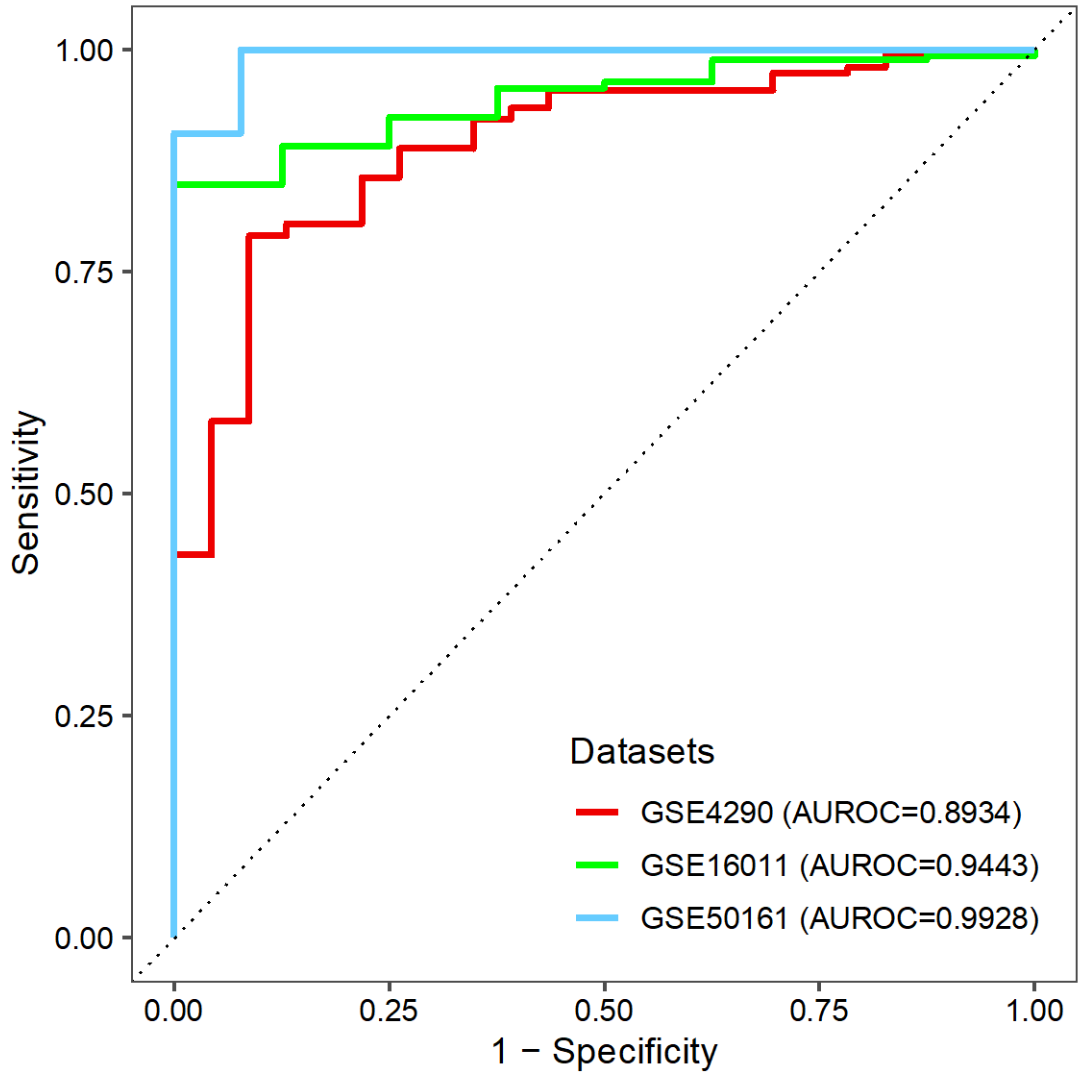

Supplementary Figure S2 Synapse scores can discriminate gliomas from normal brain tissues. ROC curves of GSE4290 (red), GSE16011 (green), GSE50161 (blue) from GEO datasets for distinguishing gliomas from normal brain tissues using synapse scores.

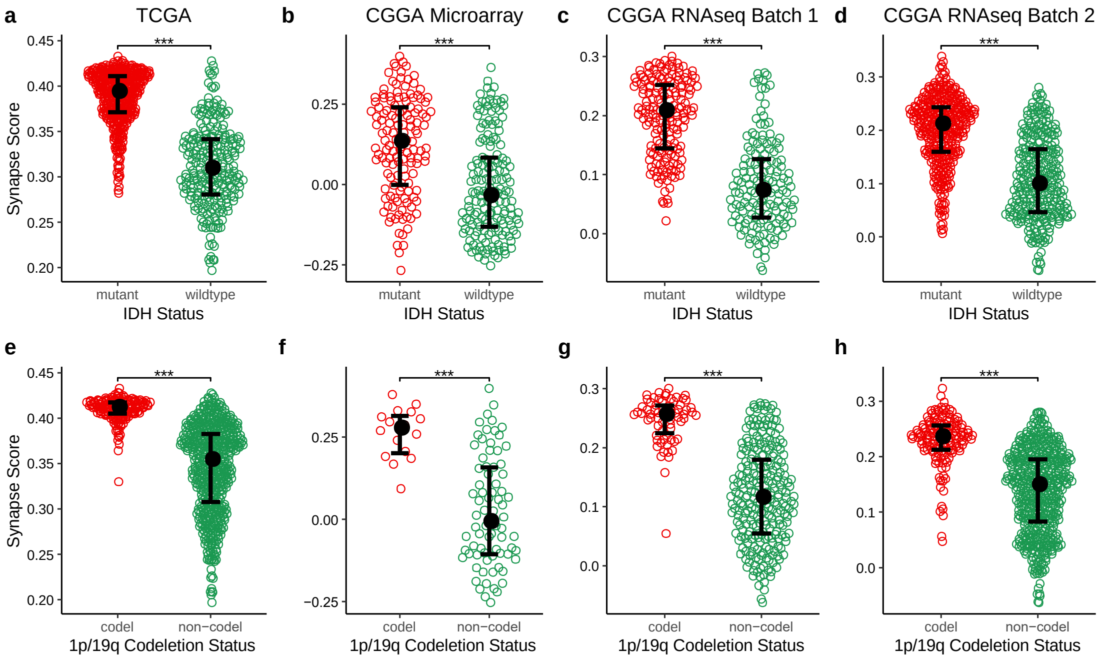

Supplementary Figure S3 Synapse scores were associated with IDH mutation and 1p/19q codeletion status. (a, e) TCGA. (b, f) CGGA Microarray. (c, g) CGGA RNAseq batch 1. (d, h) CGGA RNAseq batch 2. Significances of difference were analyzed by two-side Wilcoxon rank sum test. ** p<0.01, *** p<0.001.

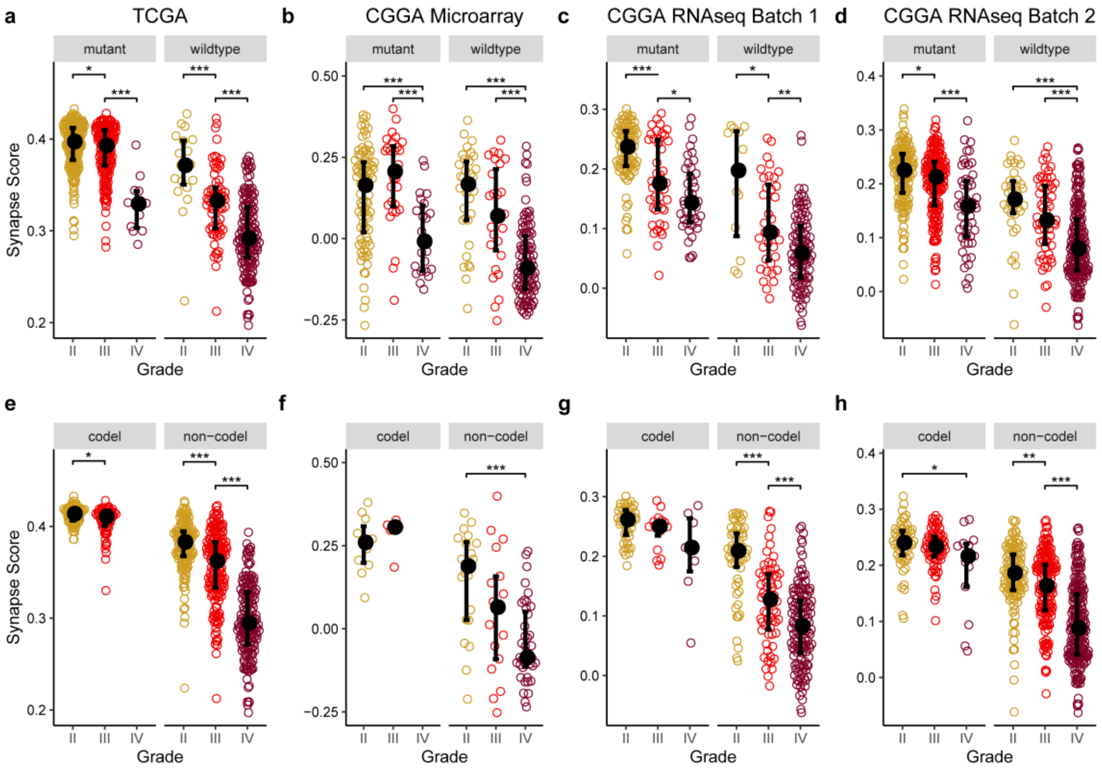

Supplementary Figure S4 The grading ability of synapse score adjusted using IDH mutation and 1p/19q codeletion. (a, e) TCGA lower grade glioma (LGG) and glioblastoma multiforme (GBM). (b, f) CGGA Microarray. (c, g) CGGA RNAseq batch 1. (d, h) CGGA RNAseq batch 2. Significances of difference were analyzed by two-side Wilcoxon rank sum test. * p<0.05, ** p<0.01, *** p<0.001.
