# Supplementary material for: A Panel of Synapse-Related Genes as a Biomarker for Gliomas
Source: Front Neurosci. 2020 Aug 11;14:822. doi: 10.3389/fnins.2020.00822 (PMC7431624; doi:10.3389/fnins.2020.00822)
Supplement: Supplementary file 2 [file Table_1.PDF]

Gene symbol	Studies in synapses/receptors/gliomas	# papers
PFN1	tumor angiogenesis[1]	51
SHANK2	NMDA receptor; AMPA receptor; autism[2; 3]	129
CACNG2	chronic pain[4; 5]	165
TNR	down-regulated in glioblastoma[6]; neuronal-specific synthesis and glycosylation[7]	85
SHISA7	AMPA receptor[8]; $GABA_A$ Receptor[9]	4
CHRNB2	nicotinic acetylcholine receptor; epilepsy[10]	120
GRIN3A	NMDA receptor; Huntington's disease[11]	61
MAPK8IP2	NMDA-mediated signal transduction[12]	13
GRID1	glutamate delta-1 receptor; spine development[13]	22
UNC13A	neurotransmission[14]	52
LRP4	glutamatergic transmission[15]; synapse formation[16]	271
SYBU	neurodegenerative diseases[17]	4
SLC16A3	resistance to hypoxia injury[18]; down-regulated in *IDH*-mut glioma[19]	15
DRP2	cholinergic synapse organization[20]	14
GRIK4	kainate receptor; imbalanced circuit output[21]	59
GRIN2C	NMDA receptor[22]	32
IGSF21	inhibitory synapse differentiation[23]	3

Supplementary Table S1 The studies of 17 selected genes in synapses, receptors, or gliomas. The numbers of papers are the numbers of results in PubMed searched by "(gene symbol) AND ((synapse) OR (receptor) OR (glioma))" (9th June 2020).

Datasets	synapse score	IDH Status	synapse score	1p/19q Status
CGGA Microarray	**1.394×10^{-11}**	6.159×10^{-10}	**3.984×10^{-8}**	4.176×10^{-5}
CGGA RNAseq batch 1	**4.649×10^{-33}**	2.859×10^{-12}	**1.414×10^{-32}**	4.156×10^{-15}
CGGA RNAseq batch 2	**1.034×10^{-32}**	6.639×10^{-29}	**1.343×10^{-34}**	9.095×10^{-14}
TCGA GBM+LGG	3.037×10^{-60}	**2.709×10^{-75}**	**5.395×10^{-61}**	1.060×10^{-13}
GSE107850	**5.702×10^{-8}**	1.633×10^{-2}	**-**	-

Supplementary Table S2 The predictive ability for survival prognosis of synapse score compared with IDH mutation and 1p/19q codeletion status. Patients were divided into two groups according to the biomarker, and the difference (p value of log-rank test) between two K-M curves was calculated. The threshold of synapse score is determined by the best separation it can perform. The better results are highlighted in bold.

References

[1] Y. Fan, A.A. Potdar, Y.Q. Gong, S.M. Eswarappa, S. Donnola, J.D. Lathia, D. Hambardzumyan, J.N. Rich, and P.L. Fox, Profilin-1 phosphorylation directs angiocrine expression and glioblastoma progression through HIF-1 alpha accumulation. Nature Cell Biology 16 (2014) 445-U144.

[2] H. Won, H.R. Lee, H.Y. Gee, W. Mah, J.I. Kim, J. Lee, S. Ha, C. Chung, E.S. Jung, Y.S. Cho, S.G. Park, J.S. Lee, K. Lee, D. Kim, Y.C. Bae, B.K. Kaang, M.G. Lee, and E. Kim, Autistic-like social behaviour in Shank2-mutant mice improved by restoring NMDA receptor function. Nature 486 (2012) 261-265.

[3] P. Monteiro, and G.P. Feng, SHANK proteins: roles at the synapse and in autism spectrum disorder. Nat Rev Neurosci 18 (2017) 147-157.

[4] J. Nissenbaum, M. Devor, Z. Seltzer, M. Gebauer, M. Michaelis, M. Tal, R. Dorfman, M. Abitbul-Yarkoni, Y. Lu, T. Elahipanah, S. delCanho, A. Minert, K. Fried, A.K. Persson, H. Shpigler, E. Shabo, B. Yakir, A. Pisante, and A. Darvasi, Susceptibility to chronic pain following nerve injury is genetically affected by CACNG2. Genome Research 20 (2010) 1180-1190.

[5] A.V. Bortsov, M. Devor, M.A. Kaunisto, E. Kalso, A. Brufsky, H. Kehlet, E. Aasvang, R. Bittner, L. Diatchenko, and I. Belfer, CACNG2 polymorphisms associate with chronic pain after mastectomy. Pain 160 (2019) 561-568.

[6] B.B. Bi, F. Li, J.S. Guo, C.L. Li, R.R. Jing, X. Lv, X.J. Chen, F.Q. Wang, K.M. Azadzoi, L. Wang, Y.G. Liu, and J.H. Yang, Label-Free Quantitative Proteomics Unravels the Importance of Rna Processing in Glioma Malignancy. Neuroscience 351 (2017) 84-95.

[7] A. Woodworth, P. Pesheva, D. Fiete, and J.U. Baenziger, Neuronal-specific synthesis and glycosylation of tenascin-R. Journal of Biological Chemistry 279 (2004) 10413-10421.

[8] L.J.M. Schmitz, R.V. Klaassen, M. Ruiperez-Alonso, A.E. Zamri, J. Stroeder, P. Rao-Ruiz, J.C. Lodder, R.J. van der Loo, H.D. Mansvelder, A.B. Smit, and S. Spijker, The AMPA receptor-associated protein Shisa7 regulates hippocampal synaptic function and contextual memory. Elife 6 (2017).

[9] W.Y. Han, J. Li, K.A. Pelkey, S. Pandey, X.M. Chen, Y.X. Wang, K.W. Wu, L.H. Ge, T.M. Li, D. Castellano, C.Y. Liu, L.G. Wu, R.S. Petralia, J.W. Lynch, C.J. McBain, and W. Lu, Shisa7 isa GABA(A) receptor auxiliary subunit controlling benzodiazepine actions. Science 366 (2019) 246-+.

[10] F. Diaz-Otero, M. Quesada, J. Morales-Corraliza, C. Martinez-Parra, P. Gomez-Garre, and J.M. Serratosa, Autosomal dominant nocturnal frontal lobe epilepsy with a mutation in the CHRNB2 gene. Epilepsia 49 (2008) 516-520.

[11] S. Marco, A. Giralt, M.M. Petrovic, M.A. Pouladi, R. Martinez-Turrillas, J. Martinez-Hernandez, L.S. Kaltenbach, J. Torres-Peraza, R.K. Graham, M. Watanabe, R. Lujan, N. Nakanishi, S.A. Lipton, D.C. Lo, M.R. Hayden, J. Alberch, J.F. Wesseling, and I. Perez-Otano, Suppressing aberrant GluN3A expression rescues synaptic and behavioral impairments in Huntington's disease models. Nature Medicine 19 (2013) 1030-+.

[12] N.J. Kennedy, G. Martin, A.G. Ehrhardt, J. Cavanagh-Kyros, C.Y. Kuan, P. Rakic, R.A. Flavell, S.N. Treistman, and R.J. Davis, Requirement of JIP scaffold proteins for NMDA-mediated signal transduction. Gene Dev 21 (2007) 2336-2346.

[13] S.C. Gupta, R. Yadav, R. Pavuluri, B.J. Morley, D.J. Stairs, and S.M. Dravid, Essential role of GluD1 in dendritic spine development and GluN2B to GluN2A NMDAR subunit switch in the cortex and hippocampus reveals ability of GluN2B inhibition in correcting hyperconnectivity.

Neuropharmacology 93 (2015) 274-284.
[14] S. Reddy-Alla, M.A. Bohme, E. Reynolds, C. Beis, A.T. Grasskamp, M.M. Mampell, M. Maglione, M. Jusyte, U. Rey, H. Babikir, A.W. McCarthy, C. Quentin, T. Matkovic, D.D. Bergeron, Z. Mushtaq, F. Gottfert, D. Owald, T. Mielke, S.W. Hell, S.J. Sigrist, and A.M. Walter, Stable Positioning of Unc13 Restricts Synaptic Vesicle Fusion to Defined Release Sites to Promote Synchronous Neurotransmission. Neuron 95 (2017) 1350-+.
[15] X.D. Sun, L. Li, F. Liu, Z.H. Huang, J.C. Bean, H.F. Jiao, A. Barik, S.M. Kim, H.T. Wu, C.Y. Shen, Y. Tian, T.W. Lin, R. Bates, A. Sathyamurthy, Y.J. Chen, D.M. Yin, L. Xiong, H.P. Lin, J.X. Hu, B.M. Li, T.M. Gao, W.C. Xiong, and L. Mei, Lrp4 in astrocytes modulates glutamatergic transmission. Nat Neurosci 19 (2016) 1010-+.
[16] A. Karakatsani, N. Marichal, S. Urban, G. Kalamakis, A. Ghanem, A. Schick, Y.N. Zhang, K.K. Conzelmann, M.A. Ruegg, B. Berninger, C.R. de Almodovar, S. Gascon, and S. Kroger, Neuronal LRP4 regulates synapse formation in the developing CNS. Development 144 (2017) 4604-4615.
[17] E. Bereczki, R.M. Branca, P.T. Francis, J.B. Pereira, J.H. Baek, T. Hortobagyi, B. Winblad, C. Ballard, J. Lehtio, and D. Aarsland, Synaptic markers of cognitive decline in neurodegenerative diseases: a proteomic approach. Brain 141 (2018) 582-595.
[18] C. Gao, W.X. Zhu, L.Z. Tian, J.K. Zhang, and Z.Y. Li, MCT4-Mediated Expression of EAAT1 is Involved in the Resistance to Hypoxia Injury in Astrocyte-Neuron co-Cultures. Neurochem Res 40 (2015) 818-828.
[19] C. Chesnelong, M.M. Chaumeil, M.D. Blough, M. Al-Najjar, O.D. Stechishin, J.A. Chan, R.O. Pieper, S.M. Ronen, S. Weiss, H.A. Luchman, and J.G. Cairncross, Lactate dehydrogenase A silencing in IDH mutant gliomas. Neuro-Oncology 16 (2014) 686-695.
[20] R.G. Roberts, and M. Sheng, Association of dystrophin-related protein 2 (DRP2) with postsynaptic densities in rat brain. Mol Cell Neurosci 16 (2000) 674-685.
[21] V. Arora, V. Pecoraro, M.I. Aller, C. Roman, A.V. Paternain, and J. Lerma, Increased Grik4 Gene Dosage Causes Imbalanced Circuit Output and Human Disease-Related Behaviors. Cell Reports 23 (2018) 3827-3838.
[22] G.L. Collingridge, R.W. Olsen, J. Peters, and M. Spedding, A nomenclature for ligand-gated ion channels. Neuropharmacology 56 (2009) 2-5.
[23] Y. Tanabe, Y. Naito, C. Vasuta, A.K. Lee, Y. Soumounou, M.W. Linhoff, and H. Takahashi, IgSF21 promotes differentiation of inhibitory synapses via binding to neurexin2 alpha. Nature Communications 8 (2017).
